# Effects of neonatal isoflurane anesthesia exposure on learning-specific and sensory systems in adults

**DOI:** 10.1038/s41598-020-70818-0

**Published:** 2020-08-14

**Authors:** Daniil P. Aksenov, Palamadai N. Venkatasubramanian, Michael J. Miller, Conor J. Dixon, Limin Li, Alice M. Wyrwicz

**Affiliations:** Department of Radiology, NorthShore University HealthSystem, 1033 University Pl., Suite 100, Evanston, IL 60201 USA

**Keywords:** Neuroscience, Risk factors, Paediatric research, Translational research

## Abstract

Millions of children undergo general anesthesia each year, and animal and human studies have indicated that exposure to anesthesia at an early age can impact neuronal development, leading to behavioral and learning impairments that manifest later in childhood and adolescence. Here, we examined the effects of isoflurane, a commonly-used general anesthetic, which was delivered to newborn rabbits. Trace eyeblink classical conditioning was used to assess the impact of neonatal anesthesia exposure on behavioral learning in adolescent subjects, and a variety of MRI techniques including fMRI, MR volumetry, spectroscopy and DTI captured functional, metabolic, and structural changes in key regions of the learning and sensory systems associated with anesthesia-induced learning impairment. Our results demonstrated a wide array of changes that were specific to anesthesia-exposed subjects, which supports previous studies that have pointed to a link between early anesthesia exposure and the development of learning and behavioral deficiencies. These findings point to the need for caution in avoiding excessive use of general anesthesia in young children and neonates.

## Introduction

Much remains unknown about the effects of commonly-used general anesthetics on the brain, particularly during development. A number of studies have indicated that exposure to general anesthetics, especially during infancy or early childhood, can affect a variety of aspects of neuronal development, leading to deficits in learning and memory later in life^1–6^. For example, it was found that children who underwent anesthesia were more than twice as likely to exhibit behavioral deficits in young adulthood^7^. The mechanisms responsible for such effects are not entirely understood, and may depend upon variables such as the duration and frequency of exposure, as well as other factors related to anesthesia delivery. As millions of children undergo general anesthesia each year during medical procedures^8^, building a better understanding of the origin and nature of these potential harmful effects of anesthesia on the developing brain has great significance from both clinical and scientific perspectives.

Studies performed in animal subjects have pointed to a variety of neuropathological changes occurring near the time of anesthesia exposure that could account for the development of the learning and memory deficits observed later in children^9^. Early anesthesia exposure has been associated, largely through histological analysis, with specific developmental abnormalities in the brain, including increased cell loss in regions such as the cerebral cortex and hippocampus which are important for learning and memory, defects in the formation of myelin, which is critical for the normal function of neurons, and disruption of learning in adult subjects^10–12^.

Previously, we used blood oxygen level dependent (BOLD) functional magnetic resonance imaging (fMRI) to examine the learning-related functional changes in response to anesthesia exposure that occur during trace eyeblink classical conditioning (ECC) in awake rabbits^13^. We found that isoflurane delivered in air severely affected the rate of ECC acquisition and produced a change in BOLD response following learning. However, the effects of neonatal anesthesia exposure have not yet been studied comprehensively using a well-controlled assessment of behavioral learning as well as structural, functional and biochemical changes at the time of young adulthood.

Here, we examined the chronic effects of isoflurane, a commonly-used general anesthetic, which was delivered to newborn rabbits. We assessed the impact of anesthesia 3 months after exposure, which is approximately equivalent to human adolescence, using MRI techniques including functional magnetic resonance imaging (fMRI), MR volumetry, spectroscopy and diffusion tensor imaging (DTI) to capture anesthesia-related changes in key learning-related brain regions, including the hippocampus and sensory cortex. Rabbits were trained with the hippocampally-dependent trace ECC paradigm in order to quantify learning rate. The circuits that underlie ECC have been well characterized^14,15^ and several studies have demonstrated the impact of early anesthesia exposure on hippocampal neurons^10,16,17^.

In addition to parameters such as the type of drug, the duration and frequency of exposure, other factors related to the delivery protocol may also play a role in anesthesia-induced pathology. One such potential factor is the level of oxygen. In a previous study^18^ we recorded a large increase of brain partial oxygen pressure during anesthesia, especially when combined with 80% oxygen, which has the potential to cause hyperoxic damage via the generation of reactive oxygen species^19^. Thus, we tested anesthesia delivery in air as well as in 80% oxygen to gauge the impact of oxygen on anesthesia-induced damage. We hypothesized the neonatal exposure to anesthesia would impair behavioral learning and produce structural, functional and biochemical changes detectable at the stage of adolescence, and that these effects would be exacerbated for anesthesia delivered in 80% oxygen.

Our results from behavioral learning measurements as well as MRI analysis demonstrated a wide spectrum of changes that were specific to anesthesia-exposed subjects, which supports previous studies that have pointed to a link between early anesthesia exposure and the development of learning and behavioral deficiencies. Specifically, functional changes were identified in the somatosensory cortex prior to learning in anesthesia-exposed subjects, and these subjects exhibited significant differences in hippocampal microstructure, volume, and metabolism compared to unanesthetized controls. Further work will be necessary to understand the mechanisms through which isoflurane and other general anesthetics interact with the brain in order to produce such effects. However, these findings suggest that care should be taken to avoid excessive use of general anesthesia in neonates.

## Results

**Figure 1:**
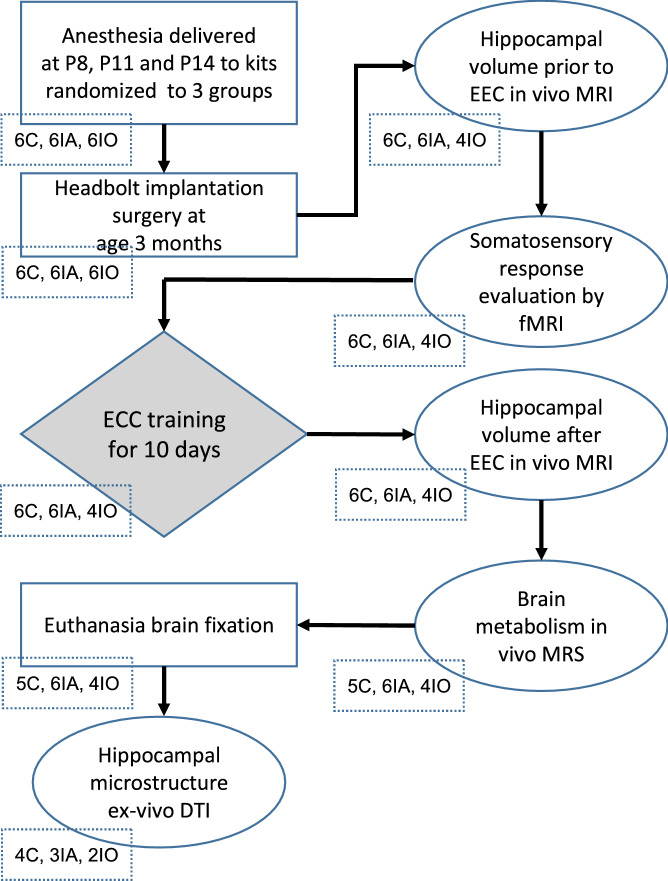
Flow chart of the experiments. Infant Dutch-belted rabbits (both sexes) were exposed to isoflurane for 2 h on P8, P11 and P14, either in air or in 80% oxygen. At the age of 3 months animals were implanted with restraining headbolts. After recovery from surgery and habituation, MR images for volumetry were acquired, followed by fMRI experiments. Subjects then received training with the trace eyeblink classical conditioning (ECC) paradigm, after which MR volumetry was repeated and followed by MRS. Finally, DTI was performed on in vitro brain preparations. Numbers of animals are indicated below each shape. *C* control, *IA* isoflurane in air, *IO* isoflurane in oxygen.

The overall experimental design included a combination of behavioral and MR tests, which were conducted before and/or after training with the trace ECC paradigm. Figure 1 summarizes the sequence of experiments that were performed for the study.

### Eyeblink classical conditioning

**Figure 2:**
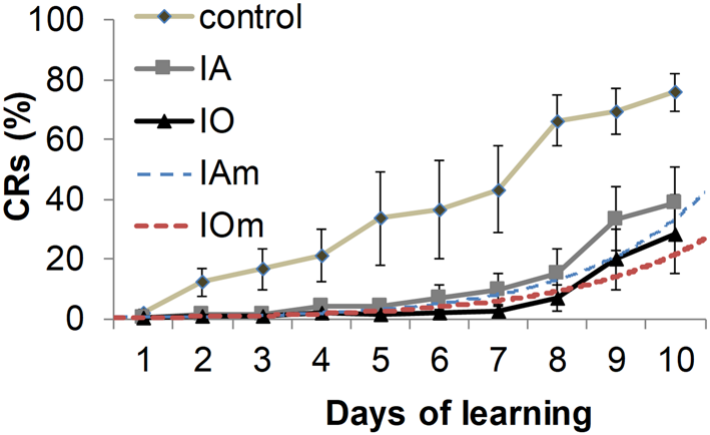
Learning curves for trace ECC are shown for the control (diamonds; N = 6), isoflurane in air (IA, squares; N = 6) and isoflurane in 80% oxygen (IO, triangles; N = 4) groups. IAm and IOm (blue and red dashed lines) represent curves generated by a non-linear regression model for the IA and IO groups, respectively. Note that by Day 10 the control group reached a mean of 70% CRs, whereas the IA and IO groups remained below 40% CRs. The control group learned at a steady rate and reached a higher level of CRs by Day 10, whereas the anesthesia groups remained below 20% CRs through Day 8. The error bars represent the SEM.

For the control group, all subjects reached the criterion level of 60% conditioned responses (CR) after training with the trace ECC paradigm. A CR is an automatic response (i.e., an eyeblink) in response to a neutral stimulus (in this case, a mechanical vibration of the whiskers). This whisker vibration is the conditioned stimulus (CS), which occurs before the airpuff, which served as the unconditioned stimulus (US). Subjects in the control group attained levels of 20% CRs after 5.2 ± 1.2 sessions, 40% CRs after 6.7 ± 0.8 sessions, and 60% CRs after 8.2 ± 0.7 sessions. Figure 2 shows the mean CR levels for each session for the control, anesthesia in air (IA) and anesthesia in 80% oxygen (IO) groups. The learning curve increased steadily for the control group, whereas mean CRs remained below 10% for the anesthesia-exposed groups until day 8. Analysis of learning levels for each session using one-way ANOVA found that a difference between the control and anesthesia-exposed groups first appeared at session 7, when the mean CRs was 43.2 ± 14.6% for the control, 9.8 ± 5.1 for anesthesia in air (IA) group, and 2.5 ± 1.5% for anesthesia in 80% oxygen (IO) group (*p* < 0.019, F = 5.5, df = 2). In session 10 the mean CRs was 75.7 ± 6.5% for the control, 39.0 ± 11.9 for IA, and 28.25 ± 13.0% for IO, (*p* < 0.012, df = 2, F = 6.44). Fisher LSD post hoc analysis showed significant differences between the control and IA (*p* < 0.015) and control and IO (*p* < 0.007) groups, but did not reveal a difference between the IA and IO groups.

In order to better capture the evolution of IA and IO learning curves over the entire duration of training, we employed a regression-based model for analysis. The estimates for the parameters and their corresponding standard errors are presented in the supplemental Table S1. The $\upgamma _{2}$ parameter estimates the difference between the learning rates of the two treatment groups. From these parameters, the following 95% confidence interval was determined:

$$ - .0863 \le\upgamma _{2} \le - .0063 $$

This confidence interval shows the statistical significance of our parameter at a five percent level. The negative value of the $\upgamma _{2}$ parameter indicates that the exponential learning rate in IO is slower than for the treatment group without oxygen. The results of the regression-based analysis are shown in Fig. 2, in comparison with the CR levels for each session. Based on this analysis, as described in the Methods section, the IA and IO learning curves were determined to be significantly different at a 95% confidence level.

### Functional MRI

**Figure 3:**
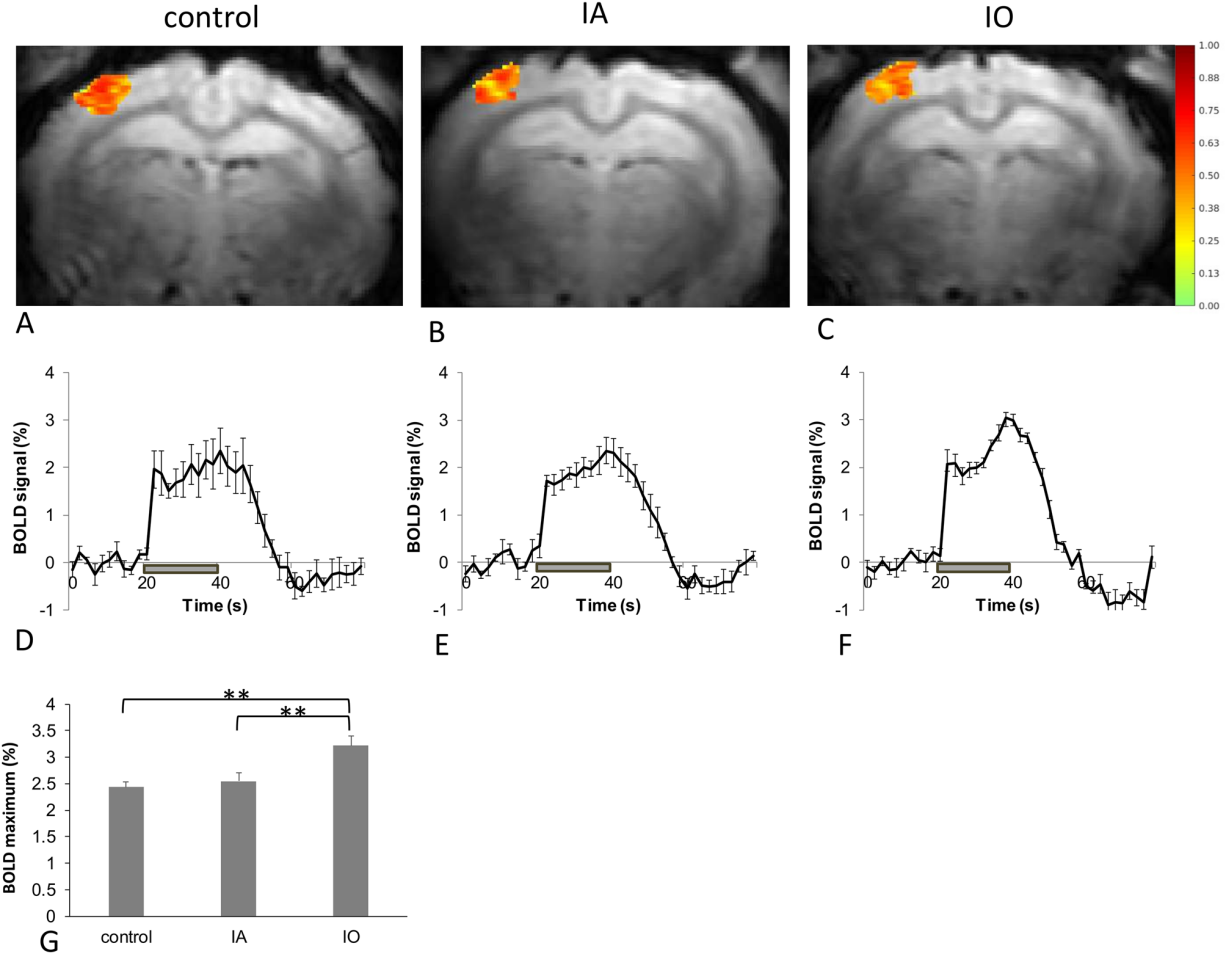
Functional responses to whisker stimulation in adolescent rabbits. Functional MRI was performed in order to evaluate the functional state of the sensory system prior to learning the trace ECC paradigm. Examples of BOLD activation in the WBC are shown from representative animals from the control (**A**) IA (**B**) and IO (**C**). In all cases rabbits exhibited a robust BOLD response which extended through all layers of the cortex. Averaged BOLD time courses for the activated region in WBC are shown for the control (**D**, N = 6), IA (**E**, N = 6) and IO groups (**F**, N = 4). BOLD activation was in the control and IA groups throughout stimulation, whereas the IO group exhibited an increase in response magnitude in the second half of the stimulus delivery. The gray bar indicates the timing of the stimulus presentation. A comparison of the averaged maximum BOLD response for each group is shown in (**G**). One asterisk represents *p* < 0.05, two asterisks represents *p* < 0.01.

BOLD fMRI data were acquired prior to learning in order to evaluate functional changes related to anesthesia exposure in the somatosensory pathway that mediates the CS. All rabbits showed a robust BOLD response to stimulation that extended through all layers of the whisker barrel cortex on the side contralateral to the stimulated whiskers. The mean BOLD activated volume for the control group was 17.5 ± 4.1 mm^3^. The mean BOLD activated volume for IA was 16.6 ± 4.4 mm^3^. The mean BOLD activated volume for IO group was 19.0 ± 3.6 mm^3^. Figure 3A–C shows representative examples of BOLD activated area. There was no statistical difference in the BOLD activated volumes between the three groups.

The average BOLD response magnitude during stimulation was 1.71 ± 0.34% for the control group, 1.76 ± 0.17% for the IA group, and 2.04 ± 0.12% for IO group. The BOLD response magnitude in control group exhibited a relatively flat shape during stimulation, without distinct peak and plateau components however, in IA group the secondary peak near the end of stimulation became visible, and was very prominent in IO group (Fig. 3D–F). To investigate further this observation, the maximum of the secondary responses were compared using one-way ANOVA (‘drug” factor with three levels: control, IA and IO) (Fig. 3G). It was revealed that “drug” factor is highly significant (*p* < 0.0092, F = 6.89, df = 2). Post hoc Fisher LSD analysis revealed that the IO group was significantly different from the control (*p* < 0.004) and IA group (*p* < 0.01).

### Hippocampal volume

Measurement of the dorsal hippocampus volume, which included regions of CA1 and dentate gyrus (DG) known to play a role in the processing of learning-related signals, allowed us to evaluate larger scale changes in hippocampal morphology associated with anesthesia exposure.

#### Before learning

**Figure 4:**
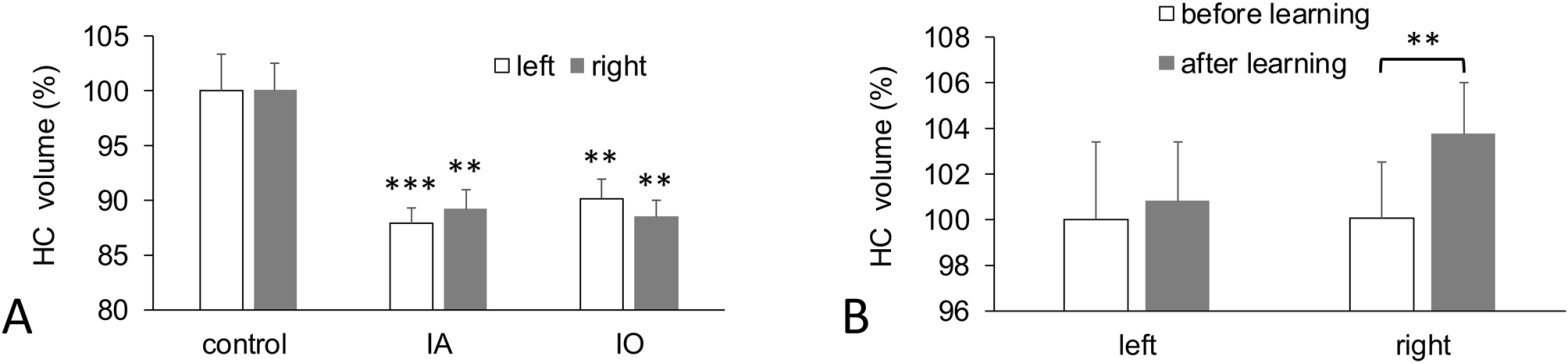
Changes in hippocampal volume. (**A**) Comparison of dorsal hippocampus (HC) volume in control (N = 6) and IA (N = 6) and IO (N = 4) groups showed that hippocampal volume in each hemisphere decreased significantly in both anesthesia-exposed groups as compared to controls. (**B**) A significant difference in volume before versus after learning on the right (i.e., learning-related) side was observed in the control group, but not in the IA or IO groups (not shown). One asterisk represents *p* < 0.05, two asterisks represent *p* < 0.01 and three asterisks represent *p* < 0.001. The values were normalized to 100% before learning.

The dorsal hippocampal volume was normalized relative to the volume of the slice containing hippocampus and expressed as a percent. The mean volume of left hippocampus in the control group was 3.9 ± 0.13% and the volume of right hippocampus was 3.9 ± 0.1%. The mean volumes of left and right hippocampus in IA were 3.42 ± 0.05% and 3.47 ± 0.06%, respectively. The mean volumes of left and right hippocampus in IO were 3.51 ± 0.07% and 3.45 ± 0.05%, respectively (Fig. 4A). Paired *t* test did not show a significant difference between left and right hippocampus in the control or anesthesia groups. Factorial ANOVA showed a significant difference for a factor “drug” (*p* < 0.001, F = 15.9, df = 2) and post hoc Fisher LSD analysis revealed that both the left and right sides of dorsal hippocampus in the IA and IO groups were significantly smaller than in the control group before learning.

#### After learning

The volume of right hippocampus in the control group increased by 3.5% after learning and paired *t* test revealed a significant difference (4.04 ± 0.09% after vs. 3.9 ± 0.1% before, *p* < 0.007) (Fig. 4B). In both the IA and IO groups, no significant differences were found before versus after learning. The mean volumes of left and right hippocampus in IA after learning were 3.51 ± 0.08% and 3.53 ± 0.07%, respectively. The mean volumes of left and right hippocampus in IO after learning were 3.49 ± 0.1% and 3.53 ± 0.1%, respectively. There was no difference in hippocampal volume between IA and IO groups as a result of learning.

### Diffusion tensor imaging

**Figure 5:**
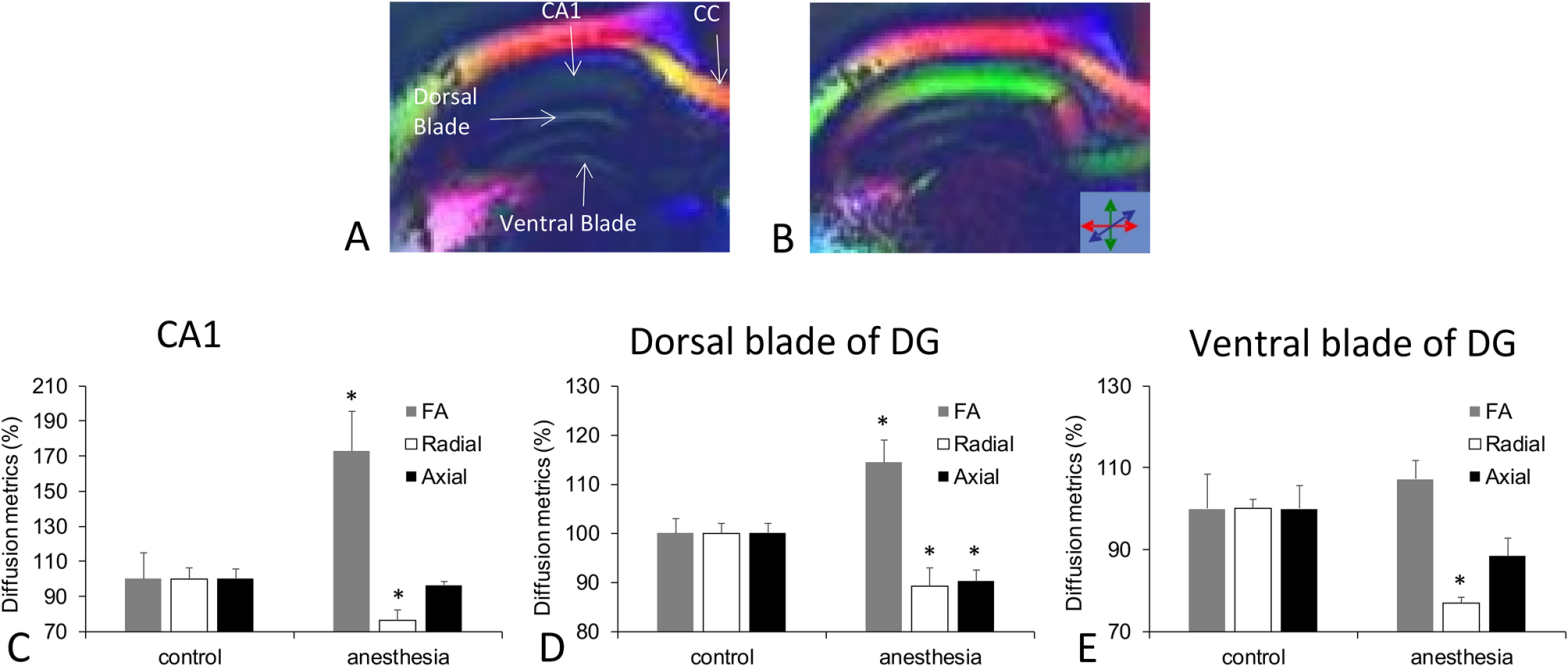
DTI of the hippocampus. DTI was used to capture the microstructural changes induced by anesthesia exposure. Fractional anisotropy (FA) maps are shown for representative subjects from the control (**A**, N = 4) and anesthesia-exposed (**B**, N = 5) groups. The color map corresponds to the direction of water diffusion. Significant diffusion changes were observed in the hippocampus in anesthesia-exposed subjects, specifically in the CA1 region and the dorsal blade of the dentate gyrus. Analysis of diffusion metrics in the control versus anesthesia groups in CA1 (**C**) revealed large increase in FA primarily arising from a decrease in radial diffusivity. In the dorsal blade of dentate gyrus (DG) (**D**), FA increased significantly as a result of decreases in both axial and radial diffusivity. No significant differences were found in the ventral blade (**E**). Lower radial diffusivity indicates less branching and lower axial diffusivity represents shorter dendritic length. CC is corpus callosum. Control values were set at 100% for these comparisons.

DTI was used to evaluate cellular organization in the CA1 and dentate gyrus regions of the dorsal hippocampus (Fig. 5A,B). We calculated fractional anisotropy (FA) and radial and axial diffusivities to obtain an overview of microstructural alterations induced by isoflurane. FA is a scalar measurement, indicating the unit value (0 to 1) of anisotropy during diffusion. A value of 1 would indicate diffusion along one axis while restricted on all other axes. A value of 0 indicates completely restricted or unrestricted diffusion along all axes. For this analysis, the IA and IO groups were combined into a single anesthesia group, as described in the Methods section. We first compared both hemispheres of the dorsal hippocampus, but paired *t* test showed that there was no difference between the right and left sides within either the control or anesthesia group. As no difference in the diffusion metrics between the two hemispheres was found, the left and right sides were averaged for subsequent ANOVA analysis to compare differences between the control and anesthesia groups.

FA in CA1 was 0.24 ± 0.04 in the control group versus 0.41 ± 0.05 in the anesthesia group which represents approximately a 60% increase in the anesthesia group. Figure 5A,B shows examples of FA maps which reveal a striking increase in FA magnitude in the CA1 region of the anesthesia group. One-way ANOVA revealed a significant difference in FA between the anesthesia and control groups (*p* < 0.038, F = 6.6, df = 1). Radial diffusivity was 0.48 ± 0.03 mm^2^/s in the control group versus 0.37 ± 0.03 mm^2^/s in the anesthesia group, corresponding to a decrease of approximately 25% in the anesthesia group. One-way ANOVA revealed a significant difference in radial diffusivity (*p* < 0.033, F = 7, df = 1) between the control and anesthesia groups, whereas axial diffusivity was not statistically different. Figure 5C shows a comparison of DTI metrics in CA1 between the control and anesthesia groups.

In the dorsal blade of DG, the mean FA was 0.22 ± 0.005 for the control group and 0.25 ± 0.009 for the anesthesia group. One-way ANOVA showed a significant difference in fractional anisotropy (*p* < 0.044, F = 6, df = 1) between the control and anesthesia groups. Radial diffusivity was 0.40 ± 0.007mm^2^/s for the control group and 0.36 ± 0.013mm^2^/s for the anesthesia group. One-way ANOVA showed a significant difference between anesthesia and control groups in radial diffusivity (*p* < 0.049, F = 5.7, df = 1). Axial diffusivity was 0.52 ± 0.01mm^2^/s for the control group and 0.47 ± 0.02mm^2^/s for the anesthesia group. One-way ANOVA showed a significant difference between anesthesia and control groups in axial diffusivity (*p* < 0.019, F = 9.2, df = 1). Thus the anesthesia group had lower axial and radial diffusivities in the DG dorsal blade. Figure 5D shows a comparison of DTI metrics in the DG dorsal blade between the control and anesthesia groups.

In the ventral blade of DG, FA and axial diffusivity were not statistically different between the control and anesthesia groups. Radial diffusivity was 0.40 ± 0.009 mm^2^/s in the control group and 0.30 ± 0.006 mm^2^/s in the anesthesia group. One-way ANOVA showed a significant difference between anesthesia and control groups in radial diffusivity (*p* < 0.0002, F = 70, df = 1). Figure 5E shows a comparison of DTI metrics in the DG ventral blade between control and anesthesia groups.

In addition to the dorsal hippocampus, DTI metrics were also analyzed in a number of white matter structures including fimbria, corpus callosum, corona radiata, internal and external capsule. No significant differences were found in the control versus anesthesia groups for any structure.

### MR spectroscopy

**Figure 6:**
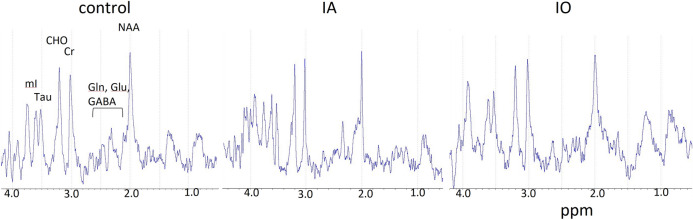
Proton MR spectra were obtained from awake adolescent rabbits. PRESS localized, water suppressed spectra from (2 mm)^3^ voxels located in the left dentate gyrus (DG) are shown. Major brain metabolites, including N-acetyl aspartate (NAA), γ-amino butyric acid (GABA), glutamate (Glu), glutamine (Gln), creatine/phosphocreatine (Cr), choline containing compounds (CHO), taurine (Tau), and myo-inositol (mI), are present in all groups. Quantification using LCModel analysis showed that all metabolite levels in the left DG were statistically similar between the control (N = 5), IA (N = 6) and IO (N = 4) groups.

MR spectroscopy was used to evaluate effects of neonatal anesthesia exposure on brain metabolism in adolescent rabbits. Proton spectra from awake rabbits acquired after learning showed characteristic resonances from *N*-acetyl aspartate (NAA), glutamate (Glu), glutamine (Gln), GABA, creatine/phosphocreatine (Cr/PCr), choline containing compounds (Cho), taurine (Tau), myo-inositol (Ins) in the DG of control and anesthesia exposed rabbits (Fig. 6).

In the left DG, there were no statistically significant differences in metabolite levels between groups. However, in the right DG, GABA was significantly higher only in the IA group compared to control group (0.11 ± 0.05 in the control group; 0.19 ± 0.05 in the IA group; 0.14 ± 0.08 in the; IO group *p* < 0.05 between control and IA groups. The levels of all other metabolites were the same between groups in the right DG also. Prompted by this observation and our finding that ECC training induced an increase in right hippocampal volume relative to left in the control rabbits, we then compared metabolite levels between left and right DG within each group. Paired *t* test revealed a significant decrease in GABA level in the right DG compared to left DG only in the control group (left 0.23 ± 0.04 vs. right 0.11 ± 0.05; *p* = 0.019). There were no differences in GABA levels between left and right DG in the IA and IO groups (IA: left 0.26 ± 0.06 and right 0.19 ± 0.05; *p* = 0.059; IO: left 0.19 ± 0.04 and right 0.14 ± 0.08; *p* = 0.319). All other metabolite levels were statistically similar between left and right DG in the three groups.

## Discussion

Previous attempts to assess the impact of early anesthesia exposure in children have been complicated by the wide range of anesthesia conditions included, which vary in duration and frequency of exposure as well as in the presence of potentially exacerbating factors such as supplemental oxygen. Animal studies allow for better experimental controls and several have examined changes in the brain near the time of anesthesia exposure. For example, it was shown that acute effects of anesthetic or antiepileptic drugs include neuronal death^20,21^ and demyelination^22^. The cell death can initiate a cascade of effects which can further disrupt physiological maturation of synapses in neurons that survive the for insults that take place during synaptogenesis^23^, leading to persistent memory/learning impairments^24^. The design of this study enabled us to capture the anesthesia-induced changes at the stage of adolescence when learning and behavioral deficits in human patients are often reported. Our findings demonstrate that adolescent rabbits previously exposed to anesthesia as neonates exhibited a variety of behavioral, functional and structural changes compared to unanesthetized subjects.

As expected, the circuits mediating trace eyeblink classical conditioning (ECC) were indeed affected by exposure to isoflurane as neonates, as indicated by the decrease in learning rate in anesthesia-exposed groups. However, as conditioned responses (CRs) were not completely abolished, the damage only partially disrupted learning-related pathways. Such deficits in learning reflect the cumulative impact of anesthesia-related damage as well as subsequent development and compensation by the brain^25^. In order to understand the nature of this damage, we used a number of MRI techniques to examine changes in key structures of the circuitry underlying trace ECC.

Normal CR acquisition depends upon an intact sensory system, and thus BOLD fMRI was performed in the somatosensory cortex, a key node in the pathway that mediates the CS, in order to compare the response to whisker stimulation of anesthesia-exposed rabbits versus un-anesthetized controls prior to training. Previous studies have demonstrated that BOLD fMRI can be used to assess sensory function for a variety of modalities, including visual deficits^26^, hearing loss^27^, and tactile and nociceptive functions^28^. Functional effects may manifest as changes in activated volume and/or shape of the BOLD response.

The consistency of activated volume among all groups indicates that anesthesia did not produce a major change in the spatial representation of the whisker stimulus in the primary sensory cortex. The BOLD hemodynamic response reflects the interaction of cortical neurons with the local vessels, and thus the preservation of this activity indicates that the neurovascular structure of the somatosensory cortex remains largely intact following neonatal anesthesia exposure.

Previously we have shown that the temporal behavior in of the BOLD response following the peak of the initial rise directly depends on the level of local inhibitory activity such that a higher level of baseline inhibition decreased the sustained BOLD magnitude during prolonged stimulation^29^. The significant change in BOLD response magnitude that we observed in the IO group indicates that somatosensation is affected by the combination of anesthesia and supplemental oxygen, and thus the deficits in behavioral learning that we observed, which were greatest in the IO group, may in part reflect altered perception of the conditioned stimulus (CS).

The differences in learning rate and functional response seen in the IO group indicate that higher-than-air oxygen concentration may be a contributing factor in anesthesia-induced learning deficiency, possibly via its impact upon excitation/inhibition within sensory pathways. Our previous findings also point to hyperoxia as a potential risk factor during anesthesia delivery. Using direct electrode measurements of oxygen concentration, we found that anesthesia delivered in 80% oxygen greatly increased (by up to 300%) oxygen concentration in the cortex of neonates^18^. It is known that the generation of reactive oxygen species directly depends on the concentration of oxygen^30^ and can lead to a variety of pathological effects in the brain^31–33^.

Previously it was assumed that sensory systems are not affected by general anesthesia in neonates. Studies in humans reported that patients up to 3 years old who were exposed to anesthesia did not develop visual acuity deficiency by age 20^34^, and full neurological examination revealed no sensory deficiency at age 8 years in children exposed to anesthesia during infancy^35^. Indeed, the sensory system is characterized by very strong regenerative abilities to compensate for even severe damage. For example, it was shown that normal visual acuity requires no more than 44% of the normal quantity of fovelar, neuro-retinal channels^36^ and that tone detection behavior was preserved even if 95% of cochlear nerve afferent synapses are damaged^37^. Here, the measurements performed in adolescent rabbits indicate that subtle but significant alterations in sensory function persist following neonatal anesthesia exposure.

In addition to sensory inputs, acquisition of trace ECC also depends upon learning-specific circuitry, and the hippocampus represents a key structure of this system. It has been well established that the dorsal hippocampus is critically important for learning trace ECC, and therefore we examined this region before and after learning in order to assess potential structural changes related to anesthesia exposure. The significant decrease in hippocampal volume in anesthesia-exposed subjects versus controls fits well with previous histological data that have shown neuronal loss in this region following anesthesia exposure^10,16,17,24^. Such damage would be consistent with the decrease in ECC acquisition rate in anesthesia-exposed groups. Notably, after learning the hippocampal volume on the learning-associated side significantly increased only in the control group, which may reflect normal learning-related hippocampal neurogenesis, angiogenesis^38,39^ and increase in dendritic branching^40^ that has been shown to occur in a variety of contexts. For example, previous MR studies have reported behavior-related changes in the hippocampus in humans after learning foreign language^41^, in exercising mice^42^, and spatial navigation training-modified hippocampal volumes in humans that protected the hippocampus against age-related volume declines^43^. The absence of such changes in the IA and IO groups after learning suggests that anesthesia-induced damage may manifest in the absence of hippocampal reorganization that normally accompanies learning.

DTI enabled us to capture a more detailed picture of the changes present in the hippocampal microstructure following neonatal anesthesia exposure. This technique measures the magnitude and direction of water diffusion^44^, and thus is sensitive to factors that affect tissue anisotropy, such as axonal density or dendritic arborization. The absence of changes in diffusion metrics in the white matter (i.e., fimbria, corpus callosum, corona radiata, internal and external capsule), indicates that axonal integrity was not severely compromised by anesthesia exposure. CA1, in contrast, exhibited a large increase in FA primarily arising from a decrease in radial diffusivity. In the dorsal blade of DG, FA increased as a result of decreases in both axial and radial diffusivity. An increase in FA, in the absence of diffusivity measurements, might be interpreted as plasticity arising from an increase in dendritic density or dendritic length. However, decreases in axial and radial diffusivities indicate that this is not the case. Lower radial diffusivity would be consistent with less dendritic branching, and lower axial diffusivity may reflect shorter dendritic length.

Together, these results indicate that neonatal exposure to general anesthesia induces chronic changes to hippocampal microstructure which could contribute to the learning deficiency that was measured at the stage of adolescence. CA1 is considered to be a critical site for associative memory and neurons in DG participate in the processing of learning-related information^45^. It has been shown previously that miR-132, one of the microRNAs responsible for positive regulation of dendritic spines was downregulated at different time points (P14, P28, P60) after neonatal rats were exposed to propofol^46^. This mechanism could be responsible for decreased dendritic length and arborization in isoflurane-exposed rabbits. In contrast to previous studies that have pointed to diffusion changes induced by learning and memory tasks^43,47^, we did not detect changes in FA which would be consistent with learning-induced dendritic remodeling in the hippocampus.

MR spectroscopy, in contrast to the DTI results, revealed only minor metabolic changes. NAA is thought to be a marker of neuronal viability^48^, and therefore maintaining the level of NAA following anesthesia exposure is an indicator that hippocampal neurons retained or recovered normal viability in the roughly 3-month interval between isoflurane exposure and the time when MRS was performed. It has been reported that NAA levels decreased immediately following brain injury but recovered to normal values within 40 days in patients with favorable neurologic outcomes^49^. Likewise, the normal level of choline suggests that cell membrane turnover is also preserved and normal level of myoinositol suggests that the cerebral osmolyte and astrocytic environment were not affected^48^.

The difference in GABA levels between right and left DG in control rabbits appears to be a consequence of learning. As described above, trace ECC is hippocampal-dependent and in our paradigm involves primarily the right hippocampus. Lower GABA in the right DG of rabbits not exposed to isoflurane corresponds to lower GABAergic inhibition in the region involved in learning. Ras-dependent increases in GABA release and increased GABAergic inhibition were found to be responsible for working memory deficits in mouse models of learning^50^. Thus, the lower differentials in GABA between the left and right hemispheres in the IA and IO groups very likely signify impaired learning in those groups compared to control animals. The findings closely parallel our ECC results, which show greater learning impairment following neonatal anesthesia exposure, particularly in the IO group.

In summary, we found a diverse array of behavioral, functional, metabolic and structural changes that persisted into adolescence following neonatal anesthesia exposure. This corresponds to the age at which such deficits are often identified in humans, but it is important to emphasize that the state of the brain at this point reflects not only the initial anesthesia-induced effects but also subsequent developmental changes and compensatory response. In future experiments, examining these effects at earlier time points would provide a clearer picture of the initial effects of anesthesia before recovery or developmental changes occur. It is possible, for example, that factors such as oxygen which were associated with more subtle changes in the sensory system and behavioral learning may produce more severe immediate damage after neonatal exposure which is obscured over time by subsequent developmental changes and reorganization/recovery. Likewise, extending behavioral and MR measurements beyond adolescence would determine whether these effects improve in adulthood or degrade further with age. For example, it would be valuable to combine MR measurements such as DTI with histological analysis at multiple time points of development in order to capture the onset of neuronal damage in greater detail including comparison between anesthesia in air and supplemental oxygen. We also note that these findings reflect the outcome of a particular anesthesia delivery protocol. Beyond the use of supplemental oxygen, additional factors such as duration, frequency of exposure and the specific drug employed likely play an important role in determining the nature and extent of anesthesia-induced damage, although further work is necessary to understand how they interact with one another. However, while much remains unknown about the mechanisms and factors that underlie these effects, our results point the need for caution in avoiding potentially excessive exposure to anesthesia during infancy and early childhood.

## Methods

### Subjects and time justification

76% of children receive a single anesthesia exposure, whereas 24% undergo multiple exposures^51^. 61% of these exposures have a duration of less than 1.5 h, with the remaining 39% lasting 1.5 h or more^51^. Thus, for this study three separate exposures of 2 h each on days 8, 11, and 14 were used to represent severe cases, based on the finding that children who underwent multiple 2-h exposures to anesthesia (due to multiple surgeries and/or MR imaging^52^) demonstrated an especially high risk of developing learning disabilities^51,53^. Rabbit brain development, including the cortical GABAergic system, occurs mostly in the perinatal period^54^, similar to humans, as opposed to altricial species like rodents or more precocial species like monkeys and sheep. The first time point of 8 days was chosen for infant rabbits because extensive myelination has been shown to start from 8 days postnatal^55^.

### Animal preparation

Dutch-belted rabbits (N = 18) were used in accordance with the National Institutes of Health guidelines and NorthShore University HealthSystem Research Institute Institutional Animal Care and Use Committee approved protocol.

For animal preparation we followed the previously published protocol^13^. The rabbit kits were born in a nest box containing shredded aspen bedding which was prepared in advance. The newborn rabbit kits were housed and nursed with the dam until 4–6 weeks, which is the optimal weaning age for Dutch Belted rabbits. Kits from each dam were randomly assigned to anesthesia and control groups. Beginning at postnatal day 8 the kits in the anesthesia group were anesthetized individually with a nose mask. Kits in the control group were exposed to the same environment as anesthesia group while breathing air. Isoflurane was administered using Vapor 19.1 vaporizers for 2hrs at 1 MAC (~ 2%) in air or in 80% oxygen with a scavenging canister on the exhaust side. The 1MAC concentration was confirmed using a tail-clamping technique. Rabbits underwent anesthesia exposures on days 8, 11, and 14 for 2 h each exposure. During anesthesia the body temperature and respiration were monitored every 15 min. A recirculating warm water heating pad (T-Pump, Gaymar Industries Inc, Orchard Park, NY, USA) was used to control temperature. The control group was placed in the same box for the same duration as anesthesia group. After each session rabbits were returned to the dam. Best efforts were made to prevent rejection of the rabbits by the dam (e.g., kits were wrapped in the nesting materials to maintain normal smell). None of the kits were rejected by the dam. Total of 18 rabbits were used. Preliminary behavioral and fMRI data from 10 rabbits in the control and anesthesia in air (IA) groups were reported previously, and were analyzed together with the additional 8 rabbits for the present study.

At the age of 3 months both female and male animals were implanted with restraining headbolts and habituated to the MRI environment as described previously^56^. Animals were anesthetized with a mixture of ketamine (60 mg/kg) and xylazine (10 mg/kg). 5–6 small bur holes were made in the skull without full penetration of the bone. Small nylon support screws were inserted into bur holes. A light-weight head restraining device containing four nylon bolts was implanted on top of the skull. This device also served to position the RF coil in the stereotaxic plane during MR experiments. The wound was completely covered with dental cement and if needed non-absorbable sutures were applied. After 1 week of recovery from surgery each subject was habituated for 3–5 days to the imaging environment prior to the experiments. 2 rabbits received anesthesia but were excluded from studies because their implant was broken. The final number of rabbits in each group for in vivo functional studies was: N = 6 (3 females and 3 males) for control, N = 6 (4 females and 2 males) for IA, and N = 4 (1 female and 3 males) for anesthesia in oxygen (IO).

### Eyeblink classical conditioning

ECC is a well-controlled test commonly used in both animals and humans in which the subject learns to associate a neutral conditioned stimulus (CS) with a behaviorally salient unconditioned stimulus (US). Rabbits were provided with earplugs during the experiments to reduce potentially distracting noise from the environment. The CS was delivered by deflecting two whiskers (A1 and B1) attached to a fiber band on the rabbit’s left side at an amplitude of 1.5 mm and a frequency of 50 Hz, using a system described previously^57^. The US consisted of a 3 psi airpuff supplied by compressed air and controlled by a regulator and solenoid valve. The US was delivered to the left eye. Eyelid movements were measured with a fiber optic-based infrared reflectance sensor^58^ which was positioned 1 cm from the cornea. The durations of the CS and US were 250 and 150 ms, respectively, with 500 ms stimulus-free trace interval. The total duration of a single trial was 8 s, with a 5–10 s random intertrial interval. A CR was defined as a change in the voltage from the detector that was 4 SD greater than the mean baseline amplitude and occurred at least 35 ms after onset of the CS but before the US. Eyeblink data were sampled at 300 Hz. Each subject received 1 session of conditioning trials per day for 10 days, where each session consisted of 100 trials. The animals were trained inside the magnet used for fMRI without the presence of noise from the pulsed field gradients. Following the training session on Day 10, the animals received 20 additional trials without attaching the whiskers to the fiber band in order to confirm that the subjects were conditioned only to the whisker stimulation and not to the sound of vibration. None of the rabbits showed more than 5% CRs for these trials.

### fMRI stimulus delivery and data acquisition

In each experiment fMRI data were acquired from awake rabbits in response to whisker stimulation. Whisker stimulation was delivered by deflecting two whiskers (A1 and B1) at an amplitude of 1.5 mm and a frequency of 50 Hz using a system described previously^57^. Each animal received 10 trials of whisker stimulation, which consisted of a baseline (30 s), stimulation (20 s) and post-stimulus (40 s) period, before and after training with the ECC paradigm.

fMRI experiments were performed as described previously^13^ using a 9.4 T imaging spectrometer (BioSpec 94/30USR, Bruker Biospin MRI GmbH) operating at 1H frequency of 400 MHz. The spectrometer was equipped with actively-shielded gradient coil (BFG-240–150-S-7, Research Resonance, Inc., Billerica, MA, USA). A single-turn, 40 mm-diameter circular RF surface coil was used for both transmission and reception. Prior to each experiment, anatomical images were acquired using a multislice gradient echo pulse sequence with a TR of 1.5 s, a TE of 10 ms, a 30 mm × 30 mm FOV, and a matrix size of 128 × 128, corresponding to an in-plane resolution of 234 µm × 234 µm. fMRI data were acquired from four consecutive in the axial plane using a single-shot, gradient-echo multi-slice EPI sequence with a repetition time (TR) of 2 s, an echo time (TE) of 11 ms, a 30 mm × 30 mm field of view (FOV), and a matrix size of 80 × 80, corresponding to an in-plane voxel size of 375 µm× 375 µm, and a 1 mm slice thickness.

The fMRI data were corrected for small head motion using a 2-D affine registration method implemented using the Insight ITK toolkit^59^ and subsequent processing was implemented in Matlab (The MathWorks, Inc., Natick, MA). Before averaging, the trials were inspected to assess the presence of any remaining head motion. Trials were then averaged and activated voxels were detected in the averaged data using an unsupervised one-class support vector machine (SVM)-based algorithm, as described previously^60^.

BOLD temporal responses were obtained by averaging the activated voxels for each region. In order to compare the temporal responses in greater detail, initial and secondary phases were defined. The initial response was defined as the maximum during the first five time points of stimulation. The secondary response was defined as the maximum during the last five points of the stimulation^29^. To compare the maxima of BOLD responses, the time courses were normalized to the average of the first five time points of stimulation to suppress individual differences in the BOLD magnitude.

### Hippocampal volume

The volumes of left and right hemispheres of the dorsal hippocampus were measured from the anatomical images obtained prior to (3 months after last anesthesia exposure) and after learning (3.5 months after last anesthesia exposure). Images were acquired using a multi-slice gradient echo pulse sequence with TR 1.5 s, TE 10 ms, 30 mm × 30 mm FOV, and matrix size 128 × 128, corresponding to an in-plane resolution of 234 µm × 234 µm. Hippocampal volume was measured from the same slice containing dorsal hippocampus at both time points (before and after learning). The brains were positioned carefully using known anatomical landmarks to represent the same slice across individual animals. The hippocampus was manually delineated in Bruker Paravision 5.1 software and the volumes of left and right hippocampus normalized to the volume of the entire slice for each animal.

### Proton MR spectroscopy

Awake rabbits were scanned on a Bruker Biospec 9.4 T imaging spectrometer. Localizer images were acquired using T_2_-weighted RARE pulse sequence. Water suppressed proton spectra were acquired from isotropic 2 mm voxels located in the left and right dentate gyrus using PRESS localization and VAPOR water suppression. TR/TE 2500 ms/10 ms, spectral width 4000 Hz, 8192 complex points and 256 averages were used. Magnetic field homogeneity was optimized on each of the selected voxels using the fast map method prior to acquiring the spectrum from that voxel. NAA, glutamate, glutamine, GABA, creatine, choline, and myo-Inositol were quantified using LC Model analysis and their levels expressed as ratio to total creatine in the same spectrum. The number of rabbits examined by MRS for each group is as follows: N = 5 (3 females and 2 males) for control (1 rabbit broke headbolt before this type of imaging and was euthanized), N = 6 for IA (4 females and 2 males) and N = 4 for IO (1 female and 3 males).

### Diffusion tensor imaging

DTI measurements were carried out ex vivo on fixed brain preparations. The 4.5 month old rabbits were deeply anesthetized with ketamine and xylazine and euthanized by perfusion with 0.9% normal saline followed by 10% formalin in phosphate buffered saline through a left intraventricular injection. The brains were subsequently post-fixed in formalin. A total of 9 brains (4 from control group (2 females and 2 males) and 5 from anesthesia group, comprising IA and IO subjects (3 females and 2 males) were examined by DTI and other brains were discarded due to damage during preparation.

For DTI, the brains were rehydrated with PBS for 48 h to remove the fixative and then positioned into a sample tube and surrounded with fomblin to prevent drying and to minimize susceptibility gradients between the brain and its environment.

DTI data was acquired on the 9.4 T imager in the axial plane with a 64-direction diffusion weighted multi-slice spin-echo imaging protocol using the following parameters: TR = 4,000 ms, TE = 24.9 ms, na = 1, one *b* value = 1,000 s/mm^2^, diffusion gradient duration δ = 7 ms, gradient separation Δ = 11.2 ms, two A0 images, number of slices 16, slice thickness 0.5 mm, FOV 34.5 mm × 45.5 mm, 256 × 192 matrix for in-plane resolution of 135 µm x 237 µm. Images were processed using Paravision 5.1 software to generate maps of fractional anisotropy (FA) and diffusivity along the x, y and z axes. The following regions of interest were manually delineated on both left and right hemispheres of the brain on the FA parameter images: dorsal hippocampus CA1 and DG (dorsal and ventral blades), fimbria, corpus callosum, corona radiata, internal and external capsule. Axial diffusivity (AD) and radial diffusivity (RD) were calculated from measured values of diffusivities in x, y and z directions. FA, AD and RD values for both hemispheres were averaged and compared between control and anesthesia groups. Since their diffusion metrics were nearly identical, the IA and IO groups were combined for analysis to increase statistical power.

### Statistical analysis

Paired t-test was used to compare pairs “before–after learning” or “left–right side” in the same animals. Factorial ANOVA (Statistica, StatSoft, Tulsa, OK) was used to determine the significance of the difference between means in anesthesia-exposed and control groups. Factorial ANOVA was used with 3 factors: drug: IA/IO/control, side: left/right, time: before/after learning. One-way ANOVA was used to compare values of diffusion tensor imaging and the differences in BOLD response between groups. The data are presented as mean + / − SEM unless otherwise specified.

In order to analyze the dynamics of the learning rates over the entire training period we applied a regression-based model, dependent on the factor of supplemental oxygen. By using least-squares regression we estimated an exponential model with three parameters: a normalizing constant, an exponential "learning rate" parameter that measures the learning rate of the air treatment group, and an interaction variable which measures the difference between the learning rates of the two treatment groups. To estimate the model we used a standard approach described in^61^ (pages 483–485) which utilizes Gauss–Newton Method (pages 472–475).

Let γ_0_, γ_1_, γ_2_ denote the three parameters described above, respectively. Our model is as follows:

$$ Y_{i} = \upgamma _{0} \exp \left( {\upgamma _{1} X_{iT} + \upgamma _{2} X_{iT} *X_{iO2} } \right) $$

where $X_{T}$ is time (in days), $X_{O2}$ is an indicator variable for the O_2_ treatment group, and $Y$ is the CR percentage. For our analysis, we primarily considered the statistical significance of the interaction term parameter ($\upgamma _{2}$). We constructed a 95% confidence interval to assess significance at the five percent level.

## Supplementary information

Supplementary Information 1.

## Data Availability

The datasets generated during and/or analyzed during the current study are available from the corresponding author on reasonable request.
