# Supplementary material for: Effects of neonatal isoflurane anesthesia exposure on learning-specific and sensory systems in adults
Source: Sci Rep. 2020 Aug 14;10:13832. doi: 10.1038/s41598-020-70818-0 (PMC7429916; doi:10.1038/s41598-020-70818-0)
Supplement: Supplementary file 1 — Supplementary Information 1. [file 41598_2020_70818_MOESM1_ESM.pdf]

Effects of neonatal isoflurane anesthesia exposure on learning-specific and sensory systems in adults

Daniil P. Aksenov, Palamadai Venkatasubramanian, Michael J. Miller, Conor J. Dixon, Limin Li, Alice M. Wyrwicz

Table S1. Estimates for the parameters of the non-linear regression model and their corresponding standard errors

k	γ_k	SE(γ_k)
0	.3741	.2670
1	.4734	.0759
2	-.0463	.0204
